# Physical activity and screen-viewing policies in Canadian childcare centers

**DOI:** 10.1186/s12889-018-6290-z

**Published:** 2019-02-04

**Authors:** Emily Ott, Leigh M. Vanderloo, Patricia Tucker

**Affiliations:** 1 Faculty of Health Sciences, University of Western Ontario, 1201 Western Road, Elborn College Rm 2580, London, ON N6G 1H1 Canada; 2 School of Occupational Therapy, University of Western Ontario, 1201 Western Road, Elborn College Rm 2547, London, ON N6G 1H1 Canada

**Keywords:** Physical activity, Screen-time, Childcare, Policy

## Abstract

**Background:**

Physical activity (PA) offers numerous health benefits for young children; however, many children enrolled in childcare engage in low levels of PA and high levels of sedentary time. This study aimed to describe the prevalence and content of written PA and screen-viewing (SV) policies in Canadian childcare centers.

**Methods:**

Using a modified version of the Environment and Policy Assessment and Observation Self-Report (EPAO-SR) tool, an online survey was distributed to all directors/administrators of center-based childcare facilities across Canada. Reminder emails were sent to encourage survey completion and a strong response rate. Descriptive statistics were used to explore demographic characteristics and frequencies were run to examine the number of centers that implemented a PA or SV policy. Deductive content analysis was completed to identify common themes in participants’ open-ended responses.

**Results:**

A total of 1158 childcare representatives participated in the study; 514 provided complete data. Of these, 295 (44%) centers indicated having a written PA policy (with the majority regulated at the provincial/territorial-level; *n* = 227; 42%). Content of these policies included amount of time: spent outdoors (*n* = 395; 63%); in teacher-led active play (*n* = 101; 16%); and PA education for children (*n* = 91; 16%). Additionally, 178 (29%) respondents reported a written policy regarding SV (with majority regulated at the center-level; *n* = 173; 34%) and primarily focusing on amount of time children watch television.

**Conclusions:**

PA regulations are more common than SV policies in Canadian childcare centers; however, less than half implement a PA policy and only a third adopt SV regulations. An opportunity exists to advance practice by adopting proactive approaches to encouraging young children to be more active and less sedentary in childcare (i.e., through written policies). Supplementing policy with accessible resources, as well as consistent provision of early childhood educator (staff) training, represent important steps for putting said policies into action.

## Background

In Canada, Early Childhood Education and Care (ECEC) is generally understood to refer to regulated programs for children from infancy to school age (up to 12 years; [1]). Since Canada does not have a federal department of education responsible for childcare or a national policy, each province and territory have constitutional responsibility for education and childcare [1]. As such, provincial and territorial jurisdictions license and monitor center-based programs for children ages 0–12 years, which typically operate on a full-day/year-round basis.

Across Canada, about 22% of children 0–5 years of age have access to regulated ECEC center-based programs, 51% are center-based spaces (ages 0–5), 35% are school-age spaces, and 14% are in family/home-based care [1]. While, provincial and territorial governments have constitutional authority for all education and childcare services and policies, the federal government primarily has responsibility for income transfers and tax credits related to ECEC (e.g., Universal Child Care Benefit, childcare income deduction; [1]). Most jurisdictions provide financial support for families who meet specific eligibility criteria (e.g., family income). However, Quebec is the only province to have a universal child daycare program, where the cost of daycare is subsidized [2].

With more than half (54%) of parents with children aged 4 and under using childcare in Canada [3], this setting provides access to a large number of children during a critical period of their growth and maturation [4, 5], and serves as a vital window of opportunity for intervention in promoting healthy behaviors. Researchers have explored the relationship between physical activity (PA) and sedentary time and the childcare environment [6, 7] and consistently noted low participation rates in PA among preschoolers attending childcare facilities [4], and high rates of sedentary time [8]. In fact, a Canadian study reported that preschoolers participated in 1.5 min/h. of moderate-to-vigorous intensity PA (MVPA; [9]), while engaging in sedentary pursuits for upwards of 42.6 min/h. in these settings [8].

A small body of research dedicated to assessing the presence/absence of formal PA and sedentary behavior (SB) policies in childcare settings and their relationship with children’s PA and SB has emerged [10–12]. Dowda and colleagues [13] reported children attending childcare settings classified as *PA promoting*, spent fewer minutes per hour in SBs and more minutes in MVPA, compared to *non-PA promoting* centers [13]. Similarly, Bower et al. [14] found children engaged in greater levels of MVPA (15% vs. 9%), and less time in SBs (50% vs. 61%) at centers with policies supporting PA opportunities. As indicated by the aforementioned research [13, 14], the presence of PA promoting policies may positively impact the PA levels in preschoolers, as well as reduce SB [10].

The newly released 24-Hour Movement Guidelines for the Early Years (0–4 year) by the Canadian Society for Exercise Physiology [15] states young children should engage in a *minimum* of 180 min of any-intensity PA per day, of which at least 60 min should be energetic play [15]. Additionally, time spent being sedentary during waking hours should be limited and screen-viewing (SV) restricted to no more than 1 h/day for children 2–4 years [15]. In comparison, for optimal health benefits children and youth (aged 5–17 years) should accumulate at least 60 min of MV PA per day and no more than 2 h of SV [15]. Despite these guidelines, PA remains an ambiguous component in Canadian childcare policies [16]. Additionally, there are limited SV guidelines explicitly outlined for early learning environments [17], therefore, it is likely childcare centers across the country vary substantially in their policies regarding PA and SV requirements.

Currently, all provinces and territories require periods of daily outdoor play pending appropriate weather conditions and provide general recommendations to afford gross motor movement [18]. However, only 3 of the 13 provinces/territories (i.e., Northwest Territories [NWT], Nunavut [NU], and Nova Scotia [NS]) provide a specific amount of time required for children to participate in PA, and only 1 province made mention of SV (i.e., New Brunswick [NB]; [18]). Given the extensive research outlining the powerful impact the environment (i.e., childcare setting) has on shaping children’s PA habits [19], it is important to recognize the potential policies may have in supporting or deterring PA participation and SB among young children within this setting [20].

Researchers suggest policy implementation within the childcare setting presents an appropriate mechanism to ensure a system-level approach to PA promotion among young children [20, 21]. However, it is important to understand what (if any) policies exist within these settings, since the frequency and components of current PA and SV policies in Canadian childcare centers remains unknown. In the absence of provincial−/territorial-regulated policies, childcare facilities across Canada may create or enforce PA and/or SV policies specific to their own center or organization. As such, the purpose of this study was to describe the *prevalence* and *content* of PA and SV policies in childcare centers across Canada. While we acknowledge SB encompasses a wide range of behaviors, for the purposes of this study, we solely focused on SV. Secondary aims of this study included describing the type of policy (i.e., written, common practice, no policy), and the governing body of these policies (i.e., province/territory, organization, center). Additionally, we explored childcare representatives’ perspectives regarding these topics.

## Methods

### Recruitment and data collection

Eligible centers were identified using each respective provinces’/territories’ online government childcare registries or as provided by a government representative. The provinces and territories of Canada are sub-national governments within the geographical areas of Canada. The major difference between provinces and territories are that provinces receive their power and authority from the *Constitution Act, 1867* (formerly called the *British North America Act, 1867*), whereas territorial governments have powers delegated to them by the Parliament of Canada.

Once approval for this study was provided by the Institution’s Research Ethics Board, the appropriate representative (i.e., program coordinator, director) at each center was contacted via email to request participation in the study (via an online, self-report survey tool in SurveyMonkey™ [22]) in August 2016. A reminder email was circulated one and three weeks later. Due to an initial low response rate from the territories (i.e., Yukon [YT], Nunavut [NU], and Northwest Territories [NWT]), maritime (i.e., Nova Scotia [NS], New Brunswick [NB], Newfoundland and Labrador [NL], and Prince Edward Island [PEI]), and prairie (i.e., Alberta [AB], Saskatchewan [SK], and Manitoba [MB]) provinces, an additional email reminder was sent, nine weeks later, to these regions only.

### Participants

#### Inclusion criteria

All *center-based* childcare facilities offering full- and part-time care to children under 5 and listed on the provincial/territorial registries, were invited to complete the survey. For the purpose of this study, the term “childcare” referred to organized group-care outside the home for children ages 2 to 5 years (e.g., nursery schools, daycare centers, church-based centers, center-based facilities, and preschool). Additionally, center-based childcare referred to any licensed early learning program for young children that provides a standardized system of care to children in a school- or institution-like setting. With removal of duplicates and bounce-back emails, the total sample of childcare representatives contacted was 7380. See Table 1 for provincial/territorial participation rates.

**Table 1:** Childcare center provincial and territorial representation and written policies (*n* = 514)

	Estimate of Total Number of Centers Across Canada (*N*)	Total Number of Centers in Sample (*N*)	Percent of Total Sample (*%*)	Centers Contacted (*n*)	Response Rate (%)	Written Physical Activity Policy (*n*)	Written Screen-Viewing Policy (*n*)
Province/Territory							
British Columbia	28,897	104	18.9	1742	6.0	32	27
Alberta	2402	31	5.6	243	12.8	14	18
Saskatchewan	310	39	7.1	201	19.4	7	6
Manitoba	349	33	6.0	223	14.8	5	3
Ontario	52,76	255	46.3	3694	6.9	144	67
Quebec	2502	34	6.2	1562	2.2	12	12
Nova Scotia	276	6	1.1	32	18.8	4	2
New Brunswick	333	26	4.7	159	16.3	11	8
Prince Edward Island	65	8	1.5	46	17.4	2	1
Newfoundland & Labrador	155	4	0.7	59	6.8	2	0
Yukon	34	2	0.4	19	10.5	1	1
Northwest Territories	892	6	1.1	51	11.8	3	3
Nunavut	27	3	0.5	25	12.0	0	0

*Note*: Some values shown in the table may not add up to 100% or *n* = 514 as some participants chose not to answer certain questions. Participation rates shown in the table may vary due to the frequency of childcare centers within each province/territory. In addition, the total number of centers included in this table are an estimate gathered from Friendly et al. Early childhood education and care in Canada 2016. Toronto: Childcare Resource and Research Unit 2016 [36]

#### Exclusion criteria

Regulated family−/home-based childcare centers, babysitters, shopping mall child-minding centers, after school programs, and programs targeting older children that were captured in the provincial registries (e.g., school-age programs) were excluded from this study. These forms of care were excluded because their policies could be quite different from center-based childcare; given differences in environment, age groups, and number of children.

### Instruments and tools

#### Tools

Using the PA and SV policy subscales, a modified Environment and Policy Assessment and Observation Self-Report (EPAO-SR) tool was administered to childcare personnel [21]. Questions regarding the PA environment (e.g., size of indoor/outdoor PA space; type of indoor/outdoor PA space; and type of fixed/portable play equipment) were also asked. Confirmation from the tool’s creators about the appropriateness of using only the policy subscale was received. To achieve our secondary aim of describing the type of policy centers were implementing (if at all), documents or written statements that are used to regulate behavior opportunities (i.e., PA and SV) were considered “policies”, whereas “common practice” described programming and/or guidelines (written or non-written) that were frequently undertaken. Either can be created and/or implemented at a local childcare center-/organizational-level and may or may not be associated with provincial-/territorial-level regulations outlined for each behavior.

The EPAO-SR is divided into three sections: Director Report, Staff Daily Questionnaire, and Staff General Questionnaire [22]. Since it was not feasible to capture information from childcare staff at facilities across the country, we used the Director Report policy subscale, with the addition of five questions from the Staff General Questionnaire (focus: equipment, environment, and space), modified to ask the director to complete. These modifications included asking the regulatory body (i.e., provincial-level, organization-level, center-level) of the policies enforced for each individual PA and SB item listed. For the purposes of this study, *provincial/territorial-level* refers to policies enforced by the respective province and territory regarding PA and SV behaviors. *Organizational-level* means a policy is created and enforced by the organization as a whole (e.g., YMCA, Head Start, etc.), and *center-level* policies refer to those created and enforced by individual childcare centers.

Additionally, two modified questions were included surrounding the childcare representative’s perceived confidence in their ability, as well as their staff’s ability to support healthy behaviors in their respective childcare centers. Only questions specific to screen-time use were asked in the SB portion of the survey.

The survey captured information pertaining to: (1) demographic information (for both the center and the staff representatives [i.e., program coordinator, director]); (2) presence and content of PA policies in their center; and, (3) presence and content of SV policies in their center. The survey also captured the directors’ receptivity of childcare centers implementing a PA policy. Open-ended questions on these topics allowed childcare representatives to expand on their responses by soliciting policy content. In addition, childcare representatives identified if the content was captured in policies (written or general practice) at their center and if so, were asked to report the regulatory body for each policy (please refer to Table 5). The online survey was circulated via SurveyMonkey™ [22], and available in English and French.

### Data analysis

Statistical analyses were conducted in SPSS (version 24). Descriptive statistics were used to explore demographic characteristics of the childcare centers and participating representatives and frequencies were run to examine the number of centers that implemented a PA or SV policy. The lower participation rate in some provinces and low presence of centers within certain provinces and territories made inter-provincial/territorial analyses not possible.

Open-ended questions were reviewed in QSR NVivo 10. Childcare response options for the EPAO-SR were grouped based on having a written policy or not, and then frequencies were explored respective to each province and territory. Similarly, for the level of regulation, childcare response options were grouped based on regulatory body enforcing policy (i.e., province/territory, organization, center) and then frequencies were explored.

Based on childcare representatives’ responses to the open-ended questions, common themes among each province and territory were examined. Specifically, the framework from the modified EPAO-SR survey, question 2 in particular, was used to determine the *parent nodes* (e.g., location, amount, type, etc.). Deductive content analysis was then carried out to identify common themes (i.e., *child nodes* [e.g., description, facilitation, licensing requirements, etc.]) in participants’ responses by examining the characteristics of the language used as well as the content or contextual meaning of the text [23].

## Results

In total, 1158 eligible childcare representatives participated in the study (response rate of 16%), with 514 providing complete data. The majority of participants (*n* = 956; 83%) represented a center-based childcare. The majority of childcare representatives reported offering care to toddlers (18 months-2.4 years; *n* = 349; 27%) and preschoolers (2.5–4 years; *n* = 425; 33%). See Tables 2 and 3 for complete participant and center demographic information.

**Table 2:** Childcare representative demographic information (*n* = 514)

	*N*	*%*
Sex		
Male	7	1.4
Female	488	94.9
Age		
20–24 years	3	0.6
25–34 years	75	14.6
35–44 years	147	28.6
45–54 years	177	34.4
55–64 years	83	16.1
65 + years	9	1.8
Ethnicity		
Caucasian	395	76.8
African Canadian	5	1.0
Aboriginal/First Nations	24	4.7
Hispanic	6	1.2
Asian	13	2.5
Arabic	4	0.8
Other	20	3.9
Position at childcare center		
Director	253	49.2
Assistant director	22	4.3
Program co-ordinator	21	4.1
Supervisor	109	21.2
Manager	51	9.9
Other	58	11.3
Years of experience in childcare setting		
< 1 year	1	0.2
1–5 years	50	9.7
6–10 years	79	15.4
11–15 years	62	12.1
16–20 years	83	16.2
21 years +	229	44.6
Educational background		
High school	2	0.4
College	283	55.2
University	173	33.7
Post-graduate degree	45	8.8
Other	10	1.9

*Note*: Some values shown in the table may not add up to 100% or *n* = 514 as some participants chose not to answer certain questions

**Table 3:** Childcare center demographic information (*n* = 514)

	*N*	*%*
Classification of childcare facility		
Center-based	956	82.5
Preschool	149	12.8
Church-based	17	1.5
Location of childcare setting		
Urban	370	72.8
Rural	138	27.2
Number of children enrolled at center		
1–44	183	35.5
45–94	207	40.1
95–134	65	12.6
135–174	24	4.7
175 +	15	2.9
Number of staff employed at center		
1–20	443	82.4
21–40	67	13.0
41+	14	2.7

*Note*: Some values shown in the table may not add up to 100% or *n* = 514 as some participants chose not to answer certain questions. Those participants who identified as home-family-based childcare facility were not eligible to complete the remainder of the survey

Most participants were employed at a childcare center in Ontario (ON; *n* = 255; 46%), followed by British Columbia (BC; *n* = 104; 19%) and prairie provinces (*n* = 103; 19%). Lower representation was achieved in the territories (*n* = 11; 2%). See Table 1 for complete provincial/territorial representation information.

### Prevalence & content of PA policies in Canadian childcare centers

Only 295 representatives (44%) indicated having a written PA policy for young children, most of which were reported in ON (*n* = 144; 49%) and BC (*n* = 32; 11%). Reported content of these policies, captured via the modified EPAO-SR tool (please refer to left-hand column of Table 4), included the amount of: time children spend outdoors (*n* = 395; 63%); teacher-led active play (*n* = 101; 16%); and PA education for children (*n* = 91; 16%). Many childcare representatives (*n* = 227; 42%) noted their PA policies as being provincially regulated. In comparison, 44 (8%) representatives cited their policies as an organizational regulation, 163 representatives (30%) indicated having center-specific policies, and 96 representatives (18%) reported having no policy with regard to PA. See Tables 4 and 5 for complete PA policy information.

**Table 4:** Prevalence and content of childcare center physical activity and screen-viewing policies

Does your center have a policy or general practice that pertains specifically to	Yes, written policy N (%)	Yes, not written policy but general practice N (%)	No N (%)
The amount of physical activity time for children	295 (44.1)	304 (45.4)	70 (10.5)
The amount of active play time for children	230 (36.5)	323 (51.3)	77 (12.2)
The amount of teacher-led active play time	101 (16.0)	329 (52.1)	202 (32.0)
The amount of time children spend outdoors each day	395 (63.0)	197 (31.4)	35 (5.6)
Appropriate clothing & shoes needed for outdoor play	436 (67.5)	174 (26.9)	36 (5.6)
Staff behavior during outdoor play time	289 (46.3)	255 (40.9)	80 (12.8)
Giving extra inside active play time as a reward	23 (3.7)	112 (17.9)	492 (78.5)
Not taking away inside active play time as a punishment	99 (15.9)	134 (21.5)	389 (62.5)
Giving extra outside play time as a reward	20 (3.2)	125 (20.1)	477 (76.7)
Not taking away outside play time as a punishment	90 (14.5)	134 (21.6)	396 (63.9)
The size of indoor active play space	171 (28.5)	185 (30.9)	243 (40.6)
The type of indoor active play space	122 (21.1)	201 (34.8)	255 (44.1)
The amount of fixed play equipment (i.e., climbers, jungle gym)	88 (14.6)	166 (27.5)	350 (57.9)
The type (i.e., climbers, jungle gym) of fixed play equipment	70 (12.2)	168 (29.2)	337 (58.6)
The amount of portable play equipment (i.e., balls, push/pull toys)	99 (16.7)	259 (43.7)	235 (39.6)
The type (i.e., balls, push/pull toys) of portable play equipment	80 (14.4)	229 (41.3)	245 (44.2)
The size of outdoor active play space	226 (40.1)	167 (29.7)	170 (30.2)
The type of outdoor active play space	139 (25.6)	172 (31.7)	231 (42.6)
The amount of time children can watch television/video each day	178 (29.3)	152 (25.0)	277 (45.6)
The type of television/video programming children are allowed to watch	135 (22.9)	141 (23.9)	313 (53.1)
The amount of time staff can spend watching television/video	79 (13.4)	104 (17.7)	406 (68.9)
The amount of time children spend working on the computer/iPad	110 (18.3)	161 (26.8)	329 (54.8)
The amount of time staff spend working on the computer/iPad	90 (15.1)	190 (31.8)	317 (53.1)
The amount of time children can play video games	94 (15.9)	114 (19.2)	385 (64.9)
The amount of time staff can play video games	69 (11.8)	92 (15.8)	423 (72.4)
Staff supervision of children’s media (e.g., television, computer, video, etc.) use	108 (18.6)	158 (27.1)	316 (54.3)
The use of media (e.g., television, computer, video, etc.) as a reward/ punishment for children	43 (7.3)	89 (15.2)	454 (77.5)
Physical activity education for children	91 (15.5)	212 (36.2)	283 (48.3)
Physical activity training for staff	55 (9.3)	193 (32.6)	344 (58.1)
Physical activity education for parents	29 (5.0)	113 (19.3)	442 (75.7)

*Note*: Some values shown in the table may not add up to 100% or *n* = 1290 as some participants chose not to answer certain questions; bolded questions indicate questions that have been qualitatively analyzed (see Tables 6, 7, 8, 9 and 10). These questions were adapted, with permission, from the Environment and Policy Assessment and Observation Self-Report tool (EPAO-SR; [21])

**Table 5:** Regulatory bodies of childcare center physical activity and screen-viewing policies

	Provincial-/Territorial-level N (%)	Organization-level N (%)	Center-level N (%)	No policy N (%)	Other N (%)
The amount of physical activity time for children	227 (42.4)	44 (8.2)	163 (30.4)	96 (17.9)	6 (1.1)
The amount of active play time for children	191 (36.0)	58 (10.9)	183 (34.5)	94 (17.7)	4 (0.8)
The amount of teacher-led active play time	50 (9.5)	75 (14.2)	221 (41.9)	172 (32.6)	10 (1.9)
The amount of time children spend outdoors each day	279 (52.5)	32 (6.0)	177 (33.3)	38 (7.2)	5 (0.9)
Appropriate clothing & shoes needed for outdoor play	40 (7.5)	103 (19.4)	343 (64.6)	39 (7.3)	6 (1.1)
Staff behavior during outdoor play time	77 (14.6)	101 (19.2)	274 (52.0)	65 (12.3)	10 (1.9)
Giving extra inside active play time as a reward	13 (2.6)	35 (7.0)	104 (20.8)	282 (56.5)	65 (13.0)
Not taking away inside active play time as a punishment	42 (8.3)	43 (8.5)	118 (23.4)	238 (47.1)	64 (12.7)
Giving extra outside play time as a reward	14 (2.8)	31 (6.2)	111 (22.0)	276 (54.8)	72 (14.3)
Not taking away outside play time as a punishment	52 (10.3)	40 (7.9)	119 (23.6)	229 (45.3)	65 (12.9)
The size of indoor active play space	253 (49.4)	36 (7.0)	106 (20.7)	100 (19.5)	17 (3.3)
The type of indoor active play space	149 (29.0)	52 (10.1)	156 (30.4)	136 (26.5)	21 (4.1)
The amount of fixed play equipment (i.e., climbers, jungle gym)	102 (20.0)	52 (10.2)	154 (30.1)	170 (33.3)	33 (6.5)
The type (i.e., climbers, jungle gym) of fixed play equipment	111 (21.7)	54 (10.6)	151 (29.5)	162 (31.7)	33 (6.5)
The amount of portable play equipment (i.e., balls, push/pull toys)	100 (19.5)	57 (11.1)	185 (36.0)	151 (29.4)	21 (4.1)
The type (i.e., balls, push/pull toys) of portable play equipment	83 (16.2)	61 (11.9)	196 (38.2)	153 (29.8)	20 (3.9)
The size of outdoor active play space	312 (61.1)	35 (6.8)	82 (16.0)	70 (13.7)	12 (2.3)
The type of outdoor active play space	181 (35.6)	53 (10.4)	136 (26.8)	120 (23.6)	18 (3.5)
The amount of time children can watch television/video each day	36 (7.1)	75 (14.9)	173 (34.3)	152 (30.2)	68 (13.5)
The type of television/video programming children are allowed to watch	35 (6.9)	72 (14.2)	180 (35.6)	150 (29.6)	69 (13.6)
The amount of time staff can spend watching television/video	20 (4.0)	61 (12.2)	149 (29.9)	186 (37.3)	83 (16.6)
The amount of time children spend working on the computer/iPad	19 (3.8)	70 (14.0)	174 (34.9)	169 (33.9)	67 (13.4)
The amount of time staff spend working on the computer/iPad	15 (3.0)	72 (14.3)	181 (36.1)	173 (34.5)	61 (12.2)
The amount of time children can play video games	19 (3.8)	61 (12.3)	156 (31.6)	177 (35.8)	81 (16.4)
The amount of time staff can play video games	12 (2.4)	59 (12.0)	136 (27.6)	191 (38.7)	95 (19.3)
Staff supervision of children’s media (e.g., television, computer, video, etc.) use	29 (5.8)	68 (13.6)	186 (37.3)	152 (30.5)	64 (12.8)
The use of media (e.g., television, computer, video, etc.) as a reward/punishment for children	23 (4.7)	52 (10.6)	136 (27.6)	188 (38.2)	93 (18.9)
Physical activity education for children	43 (8.5)	67 (13.2)	186 (36.8)	167 (33.0)	43 (8.5)
Physical activity training for staff	19 (3.7)	85 (16.8)	170 (33.5)	186 (36.7)	47 (9.3)
Physical activity education for parents	14 (2.8)	55 (11.0)	141 (28.3)	221 (44.4)	67 (13.5)

*Note*: Some values shown in the table may not add up to 100% or *n* = 1290 as some participants chose not to answer certain questions; bolded questions indicate questions that have been qualitatively analyzed (see Tables 6, 7, 8, 9 and 10). These questions were adapted, with permission, from the Environment and Policy Assessment and Observation Self-Report tool (EPAO-SR; [21])

### Childcare representatives’ descriptions of PA policies

Descriptions on the PA policies within their respective facilities were captured via the open-ended questions. Specifically, childcare centers located in QB and NB frequently reported having a regulated policy surrounding the *amount of PA* time required for children each day. Overall, the amount of PA, either regulated (i.e., policy implemented at provincial-/territorial-level, or local center-/organization-level) or common practice, varied dramatically, ranging from a minimum of 30 min to 7 h per day, and some inter-provincial differences were observed. However, the most commonly reported time was 2 h of daily outdoor playtime.

Participants also spoke about: the location of PA affordances, form of activity taking place during PA sessions, seasonal variability with respect to PA sessions, as well as PA education at their corresponding centers. See Tables 6, 7, 8, 9 and 10 for text-based examples from these questions which illustrate the major topics explored in the survey and subsequent themes that emerged.

**Table 6:** Childcare representatives’ responses about policies surrounding the amount of physical activity time at childcare centers across Canada

British Columbia	
*Licensing requires this - at least 2 h a day*	
*Guaranteed an hour of outside play time (minimum)*	
*All children will participate in 2–6 h of outdoor play and physical activity daily.*	
Alberta	
*[A]t least 6 gross motor activities per week per educator*	
*[D]aily activity outdoors up to 2 h[ours] each day*	
*[C]hildren will engage in physical activity for a minimum of 30 min every day*	
Saskatchewan	
*We follow the healthy start program which recommends 180 min or more of moving activity per day.*	
*One hour at least to run freely. Two to three guided physical activities per day*	
*[A]t least 30 min a day*	
Manitoba	
*We are outside in the playground at least once a day.*	
*1–4 h per day of outside play*	
*Average active play is 3 h per day. We use school gym only when absolutely necessary.*	
Ontario	
*Our policy does not state physical activity - it does state time outside [time] - a minimum of 2 h per day*	
*As per the CCEYA [Child Care Early Years Act], children are expected to play outdoors for a minimum of two hours per day.*	
*180 min[utes] daily of physical activity including structured and unstructured.*	
Quebec	
*2 h outdoor daily structured and unstructured*	
*3 h per day on average*	
*Children 18 months - 4 years of age should be active 180 min throughout the day.*	
Nova Scotia	
*We spend more than 50% of the day outside…*	
*[M]inimum 30 min each morning and afternoon*	
*At least 1.5 h per day*	
New Brunswick	
*We are strongly encouraged to encourage physical activity outside for at least 2 h a day.*	
*We follow the New Brunswick Operators Standards set forth by the Department of Education and Early Childhood Development which mandates a minimum of two hours daily of outside play.*	
*[M]inimum of 3 h daily*	
Prince Edward Island	
*Approx[imately]1.5 h in the [AM] then 1.5 in the afternoon.*	
*The amount of physical play happens at least 1 h in the morning (outside) and at least 1–2 h in the afternoon (outside or in the gym)*	
*The children & staff are out at least 3–4 times daily for back yard play…*	
Newfoundland & Labrador	
*On average 3 h of outdoor play per day.*	
Yukon	
*We require our children to get at least 1–2 h of physical activity daily, half will be structured and half unstructured.*	
Northwest Territories	
*20 min to 30 min of unstructured/structured outdoor/indoor.*	
*[T]he daily program plan is to include gross motor activity daily.*	
*Children receive at least 60 min per day of physical activity*	
Nunavut	
*[H]alf hour free play*	

*Note*: These responses were collected from the open-ended questions corresponding with the bolded questions in Table 4. Some provinces/territories had fewer responses, and therefore, fewer quotes were available

**Table 7:** Childcare representatives’ responses about policies surrounding location of physical activity opportunities at childcare centers across Canada

Indoor	
*[I]t is in our daily routine to have indoor physical activity inside for half an hour to one hour* (ON)	
*If it is not appropriate to go outside for the day the children will be lead in indoor running games, obstacle courses, yoga, and dancing* (YU)	
*… we encourage gross motor movements in the classrooms throughout the day* (NS)	
*We have a huge gym where the play is often unstructured* (BC)	
*We do not have access to an indoor gym but our rooms have moveable furniture so that it can be pushed aside to make a space big enough for active play* (MB)	
Outdoor	
*[M]ajority of time is outdoors…* (QB)	
*Two hours a day of outdoor play, weather permitting* (MB)	
*Children are to go outside daily* (NWT)	
*[S]pacious, variety of activity opportunities which includes climbing, crouching, crawling, planting, sliding,* etc. (NB)	
*Focus on creating ‘natural’ environments, but also have climbers, swings, and bike paths in some yards* (BC)	
Weather	
*Our policy is that we go outside daily when the weather permits it, following the CSA [Canadian Standards Association] guidelines* (BC)	
*Children are outside everyday except when the weather is bad, ex[ample] excessive rain or snow and very cold temperatures* (SK)	
*Children have 2 daily active play time outside (weather dependent) or in the gym* (NWT)	
*If weather does not permit outdoor play, an indoor gym is used for the same structure* (ON)	
*At least twice per day outside playtime weather permit* (QB)	
*All age groups are outside at least 2 times a day, in all weather* (NS)	

*Note:* These responses were collected from the open-ended questions corresponding with the bolded questions in Table 4. Location of physical activity opportunities and weather was not separated by province and territory because the responses for these topics did not present sufficient variation

**Table 8:** Childcare representatives’ responses about policies surrounding physical activity type offered at childcare centers across Canada

Structured	
*[M]inimum 45 min per week indoor structured activity (yoga, dance*; BC)	
*Two to three guided physical activities per day* (SK)	
*Structured mostly inside but some outside approx[imately]1 h a day* (PEI)	
*Teacher-led active play varies from day to day* (MB)	
Unstructured	
*[O]ur program is play based and children spend most of their day in unstructured play* (AB)	
*2 h[ours] unstructured* (NB)	
*[A]ll unstructured in/out as the children see fit* (ON)	
*We are a child led center. We do set up suggested activities; however, it is not led by a teacher* (NB)	

*Note:* These responses were collected from the open-ended questions corresponding with the bolded questions in Table 4. Physical activity type was not separated by province and territory because the responses for these topics did not present sufficient variation

**Table 9:** Childcare representatives’ responses about policies surrounding physical activity education in childcare centers across Canada

Children	
*We have visitors who share various physical activities with the children and educators talk with the children about, and role model the benefits of physical activity* (ON)	
*[A]s a play-based learning centre we try make everything a lesson* (BC)	
*Physical literacy is promoted during gross motor activities* (AB)	
*We could spend more time on teaching the children the importance of physical activity* (NS)	
Staff	
*We have designated teachers that teach Physical Education! These teachers go through 6 months of CEFA [Core Education & Fine Arts] training* (BC)	
*All staff are expected to complete on-going training in regards to all developmental areas for children* (AB)	
*Encouraged but not a requirement presently* (ON)	
*Not enough - need more resources, and cost can be a problem* (MB)	
Parents	
*We provide literature for parents about physical activity and healthy eating habits* (NB)	
*We promote all healthy habits to our parents including proper sleep, good nutrition and exercise. We do this through monthly newsletters, parent information nights, documentation and offering resources* (BC)	
*[T]his is not something that is currently implemented, but would be a great idea to educate parents on physical activity that their children need and enjoy* (ON)	

*Note:* Physical activity education was not separated by province and territory because the responses for these topics did not present sufficient variation

**Table 10:** Policies surrounding the amount and type of screen-viewing in childcare centers across Canada

Amount	
*No more than 20 min (2.5 years and up*; ON)	
*We have a policy to limit any screen time to selective times when used for educational purposes* (NB)	
*Absolutely none. We are a screen free environment* (BC)	
*Technology policy - 15 min of screen time per week/ one movie day a month* (AB)	
Computer/iPad	
*[C]hildren use computers for researching interests* (ON)	
*… only watch a dvd on the laptop on special occasions (*i.e.*, Halloween, Christmas;* NB)	
*[O]nly special needs children can use iPad for specialized apps suited to their needs or education goals, as set up by specialists* (MB)	
TV/Video	
*TV/Video usage is largely restricted and used only for educational purposes* (ON)	
*If weather is very bad we will sometimes put on a movie* (BC)	
*On special occasions only. At Christmas time we have a pajama day and we watch a movie* (QB)	

*Note:* Screen-viewing was not separated by province and territory because the responses for these topics did not present sufficient variation

### Prevalence & Content of SV policies in Canadian childcare centers

Compared to PA policies in childcare, 178 (29%) facilities identified having a written SV policy, with a majority reported in ON (*n* = 67; 38%) and BC (*n* = 27; 15%). Reported content of these policies, as captured via the modified EPAO-SR tool (please refer to left-hand column of Table 4) included: amount of time children watch television (*n* = 178; 29%); amount of time children spend working on the computer/iPad (*n* = 110; 18%); and type of television/video programming children can watch daily (*n* = 135; 23%). Most childcare centers reported having a center-specific SV policy (*n* = 173; 34%), compared to 7% (*n* = 36) cited as having policies provincially regulated, and 15% (*n* = 75) having policies dictated at the organization-level. Approximately 30% (*n* = 152) of centers reported no written SV policy. See Tables 4 and 5 for SV policy information.

### Childcare representatives’ descriptions of SV policies

The majority of centers expressed having no screens at their facility or a zero-tolerance policy. Specifically, childcare centers located in ON and BC frequently reported having a regulated policy surrounding the *amount of SV* time. Some centers specified using screens in a limited capacity (i.e., less than 20 min) or for special occasions (i.e., 2–3 times a year). For example, SV may occur on holidays and/or instances of inclement weather. In most cases, when SV is allowed, the purpose was primarily educational and supervised. Additionally, iPads or videos on the computer were used as part of a learning program suited to meet the educational goals for special needs children. Based on a review of participants’ comments, no major differences were observed regarding the type of screens adopted in the centers (i.e., computer/iPad or TV/video).

## Discussion

The first key finding of this study was that only 44% of participating childcare centers reported having written policies regarding the amount of daily PA young children receive in childcare centers. This finding is important considering previous research suggests a childcare-based policy could be an effective strategy to increase activity participation among young children [12–15].

However, it is important to recognize the mixed evidence presented in recent studies [24–26], and acknowledge that additional research is warranted to examine the varying associations these policies may have on PA within childcare centers (and the degree to which implementation adherence effects the impact on activity behaviors). Despite some contradictory findings regarding policies and their positive association on behaviors (i.e., PA and SV), recommendations and guidelines for PA and SB policies within childcare centers are emerging world-wide [27]. The findings of this study surrounding the low presence of policies align with those of Gerritsen et al. [11]. Specifically, Gerritsen and colleagues [11] reported 35% of facilities in Auckland and Waikato, New Zealand, had a written PA policy; however, no policies addressed SV within centers. Additionally, Wolfenden and colleagues [28] interviewed supervisors from licensed daycares in Australia and determined that few childcare facilities had a written PA policy (41%) [28]. Overall, these findings stress the need for policies within childcare to help engage young children in appropriate levels of PA and SB.

Despite many centers reporting daily outdoor playtime of 2 h, some childcare representatives used *required outside playtime* as a proxy for a *PA policy*. While we recognize that increased PA does occur when children are afforded more time outdoors, outdoor playtime, is not the same as a PA policy. Of the participating centers that reported having a written PA policy (44%), many acknowledged it as provincially regulated (42%). As such, it is important to note many of these provincial regulations only specify required outdoor playtime opportunities, and not the daily *amount* of PA children should be obtaining. For example, although many ON facilities reported having a PA policy, these facilities also have a mandated outdoor playtime accreditation standard [29]. While it is possible the centers have a PA policy, given the high rate at which these policies are provincially regulated, it is equally plausible childcare staff interpreted the mandated outdoor play requirement as a PA policy proxy. While research suggests outdoor active playtime is important in helping children achieve sufficient levels of PA [30, 31], this accreditation standard is not a specific policy outlining the *amount* or *intensity* of PA. It is recommended that in addition to this mandated outdoor playtime accreditation standard regulated by some provinces (i.e., NS and ON; [18]), centers implement a policy specific to the amount and/or intensity of PA children should be obtaining while in care.

Additionally, these findings align with those of Vanderloo and Tucker [18] who reviewed Canadian provincial/territorial childcare legislation regarding PA participation and discovered that only 3 provinces/territories (i.e., NWT, NU, and NS) explicitly mentioned PA in their regulations. Of these, NWT and NU provided a specific amount of time required for children to participate in activities that promote physical fitness. For the provinces/territories that had no specific time requirements for PA within their daily programming, some did mandate that children were provided with opportunities to engage in “active” or “vigorous” play or activities (i.e., BC, MB, NB, ON, PEI, QU, and YK; [18]).

Although many provinces/territories provide general PA recommendations, none provide specific time requirements for how much, how, or at what intensity [18]. Considering the lack of written PA regulations specific to childcare centers and daily PA opportunities, enforcing regulations that support but also mandate explicit PA opportunities (i.e., how much, how, or intensity-specific) could play a significant role in ensuring children are obtaining the recommended amount of daily activity [18].

In terms of SB, only 29% of participating childcare centers identified having a written SV policy. Despite few centers employing SV-specific policies, the majority reported having no screens at their facilities. In some instances, SV pursuits were described as limited or used in a teaching capacity; however, a recent review has shown that with higher engagement in SBs (e.g., access to computers, television, and videos) children spend less time in MVPA [9]. If SV behaviors are not regulated, they have the potential to displace beneficial PA. These findings are consistent with previous research, suggesting SB policies, specifically SV regulations, are limited within childcare centers [10, 18]. Specifically, Vanderloo and Tucker [18] reported only 1 out of 13 provinces and territories have a regulation that states that television viewing should not be a part of the children’s daily programming during care hours. Despite sometimes serving educational purposes, screen-time should be supervised and limited in accordance to the Canadian guidelines (i.e., no more than 1 h/day for children 2–4 years; [15]).

Research has emphasized the strong influence childcare providers can have through educating and role-modelling healthy behaviors for children in their care [32]. Qualitative analyses identified that although many childcare representatives recognized the importance of PA education for staff as well as the children, the cost or lack of resources was a barrier to providing this service. These comments echo those in previous research acknowledging the need for additional staff resources and training to implement PA with preschoolers [28, 33]. The adoption of comprehensive policies regulating the amount and type of PA education opportunities for early childhood education students as well as staff, may be another strategy to support these environments and ensure young children are receiving adequate PA and SB programming [34].

With few provincial/territorial PA and SV policies, specific to amount, frequency, and type of PA/SV implemented in Canadian childcare centers, variability likely exists surrounding affordances for each behavior, as each childcare center may interpret PA and SV opportunities differently. More specifically, the onus is on childcare centers to implement their own policies regarding PA and SV. As such, it is important that childcare stakeholders are aware of the Canadian 24-Hour Movement guidelines to ensure appropriate opportunities are being offered [15]. Additionally, it is important to highlight that supplementing policy with accessible resources (e.g., posters, information sheets about PA and SV, etc.), as well as consistent provision of early childhood educator (staff) training, may represent important steps for putting said policies into action [15, 20, 34].

### Strengths and limitations

Strengths of this study include a large sample of childcare representatives from across Canada as well as the use of a widely-recognized and valid tool aimed at exploring the PA and SB environment within childcare centers [21]. However, this research only collected self-reported information from childcare representatives, rather than conducting an environmental scan of childcare policy documents. Not only could social desirability bias result because of this, there is also no evidence available to know how well (or not) the policy is implemented and followed by staff in respective childcare facilities. Second, Canada has two official languages, and to maximize participation, the EPAO-SR was translated into French (a noted strength); however, no reliability or validity scores are available for this version of the tool. While every effort was made to invite all center-based childcare centers in Canada to participate and in turn, encourage survey completion, authors were only able to achieve a response rate of 16%, slightly below the average response rate of 23% for online-based survey research [35]; therefore, limiting the overall generalizability of these findings. While participation was secured from childcare centers in every province and territory (although higher rates in some provinces), we are unable to confirm the representativeness of our sample, as no national-level data on childcare characteristics is available. We have provided details regarding our sample in Table 3 to provide context to the study findings. Finally, while a low report of written policies was observed in this study, most responses were collected from ON (*n* = 255; 46%); it is important to note ON has the most childcare centers in the country, and as a result, accounted for half of all centers contacted in this study.

## Conclusions

This study is the first of its kind to provide evidence of the PA and SV policy landscape in Canadian childcare facilities. Considering the limited provincial/territorial childcare regulations and accreditation standards, the results of this study provide preliminary, yet important insights into better understanding the frequency of PA and SV policies in childcare settings. Future efforts are warranted to continue to understand the impact of policy on young children’s PA and SV behaviors in a larger, more representative sample.

## Abbreviations

AB: Alberta
BC: British Columbia
CCEYA: Child Care Early Years Act
CEFA: Core Education & Fine Arts
ECEC: Early Childhood Education and Care
EPAO-SR: Environment and Policy Assessment and Observation Self-Report
MB: Manitoba
MVPA: Moderate-to-Vigorous Physical Activity
NB: New Brunswick
NL: Newfoundland & Labrador
NS: Nova Scotia
NU: Nunavut
NWT: Northwest Territories
ON: Ontario
PA: Physical Activity
PEI: Prince Edward Island
QB: Quebec
REB: Research Ethics Board
SB: Sedentary Behavior
SK: Saskatchewan
SV: Screen-Viewing
TV: Television
YU: Yukon

## References

[1] Howe N, Flanagan K, Perlman M. Early childhood education in Canada. In M. Fleer, B. van Oers (Eds.) International Handbook on Early Childhood Education and Care, Volume 1. Netherlands: Springer; 2017.

[2] Stalker G, Ornstein M (2013). Quebec, daycare, and the household strategies of couples with young children. Can Public Policy.

[3] Sinha M (2014). Spotlight on Canadians: results from the general social survey–child Care in Canada.

[4] Hinkley T, Salmon J, Okely AD, Hesketh K, Crawford D (2012). Correlates of preschool children’s physical activity. Am J Prev Med.

[5] Garriguet DCV, Colley RC, Janssen I, Timmons BW, Tremblay MS. Physical activity and sedentary behaviour of Canadian children aged 3 to 5. Health Reports, Statistics Canada. 2016;27(9):14–23. 27655168

[6] Razak LA, Yoong SL, Wiggers J, Morgan PJ, Jones J, Finch M (2018). Impact of scheduling multiple outdoor free-play periods in childcare on child moderate-to-vigorous physical activity: a cluster randomised trial. Int J Behav Nutr Phys Act.

[7] Peden ME, Jones R, Costa S, Ellis Y, Okely AD (2017). Relationship between children’s physical activity, sedentary behavior, and childcare environments: a cross sectional study. Prev Med Rep.

[8] Tucker P, Vanderloo LM, Burke SM, Irwin JD, Johnson AM (2015). Prevalence and influences of preschoolers’ sedentary behaviors in early learning centers: a cross-sectional study. BMC Pediatr.

[9] Vanderloo LM, Tucker P, Johnson AM, van Zandvoort MM, Burke SM, Irwin JD (2014). The influence of Centre-based childcare on preschoolers’ physical activity levels: a cross-sectional study. Int J Environ Res Public Health.

[10] Erinosho TO, Hales DP, Vaughn AE, Mazzucca S, Ward DS. Physical activity policies at childcare centers and impact on children’s physical activity and screen-time behaviors. The FASEB Jouranal. 2013;27(1):236.

[11] Gerritsen S, Morton SM, Wall CR (2016). Physical activity and screen use policy and practices in childcare: results from a survey of early childhood education services in New Zealand. Aust N Z J Public Health.

[12] O’Neill JR, Dowda M, Benjamin Neelon SE, Neelon B, Pate RR (2017). Effects of a new state policy on physical activity practices in child care centers in South Carolina. Am J Public Health.

[13] Dowda M, Brown WH, McIver KL, Pfeiffer KA, O'Neill JR, Addy CL, Pate RR (2009). Policies and characteristics of the preschool environment and physical activity of young children. Pediatrics.

[14] Bower JK, Hales DP, Tate DF, Rubin DA, Benjamin SE, Ward DS (2008). The childcare environment and children’s physical activity. Am J Prev Med.

[15] Tremblay MS, Chaput J-P, Adamo KB, Aubert S, Barnes JD, Choquette L, Duggan M, Faulkner G, Goldfield GS, Gray CE (2017). Canadian 24-hour movement guidelines for the early years (0–4 years): an integration of physical activity, sedentary behaviour, and sleep. BMC Public Health.

[16] Physical activity in Canadian early childhood education and care. http://www.childcarecanada.org/resources/issue-files/physical-activity-canadian-early-childhood-education-and-care. Published 2013. Accessed 9 Nov 2016.

[17] Vanderloo LM (2014). Screen-viewing among preschoolers in childcare: a systematic review. BMC Pediatr.

[18] Vanderloo LM, Tucker P (2018). Physical activity and sedentary behavior legislation in Canadian childcare facilities: an update. BMC Public Health.

[19] Goldfield GS, Harvey A, Grattan K, Adamo KB (2012). Physical activity promotion in the preschool years: a critical period to intervene. Int J Environ Res Public Health.

[20] Vanderloo LM, Tucker P, Ismail A, van Zandvoort MM (2012). Physical activity opportunities in Canadian childcare facilities: a provincial/territorial review of legislation. J Phys Act Health.

[21] Ward DS, Mazzucca S, McWilliams C, Hales D (2015). Use of the environment and policy evaluation and observation as a self-report instrument (EPAO-SR) to measure nutrition and physical activity environments in child care settings: validity and reliability evidence. Int J Behav Nutr Phys Act.

[22] SurveyMonkey Inc.™ (2015) San Mateo, California, USA. www.surveymonkey.com.

[23] Hsieh H-F, Shannon SE (2005). Three approaches to qualitative content analysis. Qual Health Res.

[24] Benjamin Neelon SEFJ, Neelon B, Gillman MW (2017). Evaluation of a physical activity regulation for child care in Massachusetts. Child Obes.

[25] Copeland KA, Khoury JC, Kalkwarf HJ (2016). Child care center characteristics associated with preschoolers’ physical activity. Am J Prev Med.

[26] Erinosho T, Hales D, Vaughn A, Mazzucca S, Ward DS (2016). Impact of policies on physical activity and screen time practices in 50 child-care centers in North Carolina. J Phys Act Health.

[27] Pate RR, O’neill JR (2012). Physical activity guidelines for young children: an emerging consensus. Arch Pediatr Adolesc Med.

[28] Wolfenden L, Neve M, Farrell L, Lecathelinais C, Bell C, Milat A, Wiggers J, Sutherland R (2011). Physical activity policies and practices of childcare centres in Australia. J Paediatr Child Health.

[29] Child Care and Early Years Act, Amended to O. Reg. 137/15 GENERAL. https://www.ontario.ca/laws/regulation/150137. Published 2014. Accessed Jan 2017.

[30] Truelove S, Bruijns BA, Vanderloo LM, O'Brien KT, Johnson AM, Tucker P. Physical activity and sedentary time during childcare outdoor play sessions: a systematic review and meta-analysis. Prev Med. 2018. 10.1016/j.ypmed.2017.12.022. 10.1016/j.ypmed.2017.12.022 29305869

[31] Vanderloo LM, Tucker P, Johnson AM, Holmes JD (2013). Physical activity among preschoolers during indoor and outdoor childcare play periods. Appl Physiol Nutr Metab.

[32] Copeland KA, Kendeigh CA, Saelens BE, Kalkwarf HJ, Sherman SN (2011). Physical activity in child-care centers: do teachers hold the key to the playground?. Health Educ Res.

[33] Tucker P, van Zandvoort MM, Burke SM, Irwin JD (2011). Physical activity at daycare: childcare providers’ perspectives for improvements. J Early Child Res.

[34] Martyniuk OJ, Tucker P (2014). An exploration of early childhood education students’ knowledge and preparation to facilitate physical activity for preschoolers: a cross-sectional study. BMC Public Health.

[35] Nulty DD (2008). The adequacy of response rates to online and paper surveys: what can be done?. Assess Eval High Educ.

[36] Friendly M, Larsen E, Feltham LE, Grady B, Forer B, Jones M (2016). Early childhood education and care in Canada 2016.

